# Complexity of Cardiovascular Regulation and Its Association with Physical and Cardiorespiratory Fitness in Men with Type 2 Diabetes Mellitus

**DOI:** 10.3390/healthcare14070940

**Published:** 2026-04-03

**Authors:** Étore De F. Signini, Raphael M. de Abreu, Alex Castro, Andréia M. Santos, Gabriela A. M. Galdino, Silvia C. G. Moura, Stephanie N. Linares, Juliana C. Milan-Mattos, Rafaella M. Zambetta, Alberto Porta, Aparecida M. Catai

**Affiliations:** 1 Department of Physiotherapy, Universidade Federal de São Carlos, São Carlos 13565-905, Brazil; 2 Department of Health, LUNEX University of Applied Sciences, 4671 Differdange, Luxembourg; 3 LUNEX ASBL Luxembourg Health & Sport Sciences Research Institute, 4671 Differdange, Luxembourg; 4 Brazilian Biosciences National Laboratory, Brazilian Center for Research in Energy and Materials, Campinas 13083-100, Brazil; 5 Department of Biomedical Sciences for Health, University of Milan, 20133 Milan, Italy; 6 Department of Cardiothoracic, Vascular Anesthesia and Intensive Care, IRCCS Policlinico San Donato, 20097 San Donato Milanese, Italy

**Keywords:** cardiovascular system, metabolic diseases, exercise test, oxygen consumption, humans

## Abstract

**Background/Objectives**: Cardiovascular regulation complexity (CRC) is an underexplored health marker in the context of type 2 diabetes mellitus (T2DM). Additionally, associating CRC with physical and cardiorespiratory fitness variables could provide greater insight into how physical conditioning impacts cardiovascular health in the context of T2DM. This study aims to investigate whether the relationship between physical and cardiorespiratory fitness and CRC differs according to the presence or absence of T2DM. **Methods**: Sixty-eight men were equally divided into the T2DM group (T2DMG; 57 ± 6 years old and 28.4 ± 3.1 kg/m^2^) and the control group (CG; 52 ± 5 years old and 25.1 ± 2.8 kg/m^2^). Participants underwent a resting cardiovascular data collection and a cardiopulmonary exercise test on a cycle ergometer. For each group, the relative peak power (W/kg_PEAK_) and peak oxygen consumption (VO_2PEAK_) were correlated with the CRC indices, namely, Shannon entropy, the complexity index, the normalized complexity index, and the sample entropy from heart period (HP) and systolic arterial pressure (SAP) series. A partial correlation was performed for each group, controlling for age, physical activity level, and metabolic cart. **Results**: Only the CG showed positive and significant correlations between relative VO_2PEAK_ and W/kg_PEAK_ and CRC indices derived from the HP series (0.354 ≤ *r* ≤ 0.548 and 0.001 ≤ *p* ≤ 0.047). Correlations with the SAP series were not significant, regardless of the groups. **Conclusions**: In this sample, there was no positive relationship between physical and cardiorespiratory fitness variables and CRC indices among individuals with T2DM. Further large sample studies are needed to elucidate the factors involved in T2DM that impact CRC.

## 1. Introduction

Type 2 diabetes mellitus (T2DM) is an emerging health problem due to its increasing number of cases over the years [1]. The increased prevalence of this condition is often related to unhealthy lifestyle habits (such as sedentary behavior and imbalanced diet) resulting from environmental and social changes inherent in society’s development, population aging, and the lack of support policies in sectors such as health, agriculture, transportation, food processing, and education [2,3,4]. Additionally, T2DM involves impaired insulin production, chronic hyperglycemia, and often plays a role in the genesis and progression of potentially fatal or nonfatal debilitating cardiovascular diseases [5,6,7].

Cardiovascular and metabolic diseases are typically linked to impaired regulatory mechanisms [8,9]. The cardiovascular system plays an important role in the body, focusing especially on supplying vital functions and aiding in the elimination of waste products [10]. It is composed of highly specialized and complex subsystems that interact with each other and central neural commands to accomplish tasks [10]. This interaction aims to regulate the cardiovascular system to maintain homeostasis and meet the body’s energy demands [11,12]. Cardiovascular regulation is an incessant process, and its complexity is directly related to the integrity of the subsystems and the quality of interactions between them [10]. Therefore, cardiovascular regulation complexity (CRC) serves as an indicator of cardiovascular health, which may decrease in pathological conditions [10]. This makes the study of the connection between T2DM and CRC promising, since it might show how T2DM affects the complexity of autonomic regulations and their interaction above and beyond the well-known impact of T2DM on autonomic function.

Previous studies by our group have shown that T2DM alone does not significantly alter some indices of CRC under resting conditions [13]. However, little is known about the influence of T2DM on CRC in the context of physical fitness. The T2DM impairs cardiorespiratory fitness (CRF) and physical fitness primarily through the induction of changes of myocardiogenic, myogenic, vasogenic, and neurogenic origin [14,15]. Nevertheless, regular physical exercise improves physical fitness, reduces the negative effects of T2DM, improves health conditions, and enhances the functionality of patients affected by this clinical condition [16]. Although this is well-established, the relationship between fitness level and CRC in the context of T2DM remains largely unknown.

A greater level of physical fitness is expected to result from greater systemic integrity and, therefore, may be associated with greater CRC. This speculation becomes intriguing in the context of T2DM since cardiometabolic impairment is already involved. Therefore, it is important to evaluate the relationship between CRC and physical fitness in the context of T2DM. The results of this study may contribute to a better understanding of how physical conditioning affects the cardiovascular health of individuals with T2DM. Thus, this study aimed to investigate whether the relationship between physical and cardiorespiratory fitness and CRC differs according to the presence or absence of T2DM.

## 2. Materials and Methods

### 2.1. Subjects

A total of 68 men, aged 45 to 70 years, were evaluated. The participants were divided equally into two groups: individuals who were apparently healthy (control group (CG)) and individuals with T2DM (type 2 diabetes mellitus group (T2DMG)), as classified by the American Diabetes Association [17]. The T2DMG included individuals without diagnosed inflammatory, cerebrovascular, cardiovascular, or respiratory disease not directly related to T2DM, and without mobility limitations. Additionally, they were not regular alcohol consumers, had been smoke-free for at least one year, and did not use medications (such as beta-blockers) that directly act in cardiovascular autonomic modulation. The CG consisted of individuals with the same characteristics, but without T2DM. Individuals with significant cardiovascular abnormalities observed during the protocol, such as signs of myocardial ischemia (ST segment depression), excessive arrhythmias, blood pressure hyperreactivity, or recurrent hypotension, were excluded, as well as individuals with blood test results suggesting other significant diseases (such as leukemia).

This study was approved by the Human Research Ethics Committee at the Federal University of São Carlos (UFSCar) (numbers: 6.848.846 and 3.852.163) and conducted in accordance with the standards set by the Declaration of Helsinki. All participants signed a free and informed consent form after agreeing to participate in this study.

### 2.2. Experimental Design

After agreeing to participate in this study, the participants underwent tests and assessments. These were divided into two days with a short interval between them (≤14 days). On the first day, the participants had their resting cardiovascular data collected. On the second day, they performed the cardiopulmonary exercise test (CPET). The participants also underwent a fasting blood test at a specialized laboratory in São Carlos. The test was performed after 10 to 12 h of fasting to assess their health condition.

For all tests and assessments, individuals were instructed to avoid strenuous physical activity on the day before and on the day of the assessments. They were also instructed to abstain from consuming food and beverages containing alcohol or stimulants (e.g., coffee, energy drinks, and foods with high amounts of added sugar) for 24 h before the tests and assessments. Additionally, they were advised to get a proper night’s rest the night before and not arrive fasting (excluding for blood tests) or immediately after a meal. Participants received text messages or phone reminders about these instructions in the days before the assessments and were asked about their adherence to the instructions on assessment days. The two days of evaluation performed at the cardiovascular physiotherapy laboratory (LFCV) of UFSCar were conducted in rooms with controlled environmental conditions. The environmental temperature was maintained between 21 °C and 24 °C, and the relative humidity was kept between 40% and 60% [18].

### 2.3. Cardiovascular Data Collection

After stabilizing the cardiovascular variables in the supine position for 10 min, the electrocardiogram (ECG) and continuous noninvasive blood pressure were collected continuously for 15 min at rest. During this time, participants were instructed to remain still, silent, and awake.

The ECG signals were acquired via MC5 lead (BioAmp FE132, ADInstruments, Bella Vista, NSW, Australia), and the arterial pressure was acquired using non-invasive finger photophethysmography (Finometer Pro, Finapres Medical Systems, Enschede, The Netherlands). Analog-to-digital conversion was carried out by the acquisition device Power Laboratory 8/35 hardware (ADInstruments, Bella Vista, NSW, Australia) and analyzed with the LabChart software, version 7.3.8 (ADInstruments, Dunedin, New Zealand).

### 2.4. Cardiopulmonary Exercise Test (CPET)

The CPET was performed on a cycle ergometer (CORIVAL V3, Lode BV, Groningen, The Netherlands) using a ramp protocol. The increment calculation was based on the Wasserman equation [19] with adaptations, according to the evaluator’s experience [20], for more physically active individuals. The CPET consisted of a 3 min warm-up with no added load, followed by 8 to 12 min of ramp increment and 6 min of active recovery. If the load increase was either overestimated or underestimated, leading to a ramp increment time of less than 8 min or more than 12 min, respectively, the evaluation was rescheduled for another day. The revolutions per minute were kept between 60 and 70 during warm-up, increment, and active recovery. The test was conducted until exhaustion or the occurrence of a termination criterion, according to Balady’s proposal [21]. The peak oxygen consumption (VO_2PEAK_) was calculated using the average of the last 30 s, and the peak power (W_PEAK_) was the highest value achieved during the load increment phase. In this study, the variables of greatest interest obtained in the CPET were the relative VO_2PEAK_ and W_PEAK_, expressed as VO_2PEAK_ (mL/kg/min) and W/kg_PEAK_, respectively, as these variables provide precise information about CRF and physical fitness. All patients undergoing CPET were attended to by a cardiologist.

Ventilatory and metabolic variables were obtained, breath-by-breath, through a metabolic cart (ULTIMA MedGraphics, St. Paul, MN, USA) and processed using specific software (Breeze Suite 7.1, MedGraphics, St. Paul, MN, USA). The 12-lead ECG was obtained by an electrocardiograph (CardioPerfect, Welch Allyn, New York, NY, USA). A metabolic cart VMAX (Encore, Carefusion, Yorba Linda, CA, USA) and the 12-lead ECG obtained by WinCardio System (WinCardio^®^ System, Micromed, Brasília, Brazil) were also used.

### 2.5. CRC

To perform the CRC analysis, sequences of 256 consecutive heart period (HP) and systolic arterial pressure (SAP) values were selected from the most stable regions of the tachogram and systogram [18,22,23], respectively, as defined by visual inspection [18]. The HP and SAP values were carefully checked to avoid erroneous detections or missed beats due to alterations in R-wave and blood pressure delineations before sequence selection [18]. Isolated ectopic beats were corrected using linear interpolation with unaffected adjacent HP and SAP values [24]. We opted to evaluate CRC using indices derived from the HP and SAP series to assess the cardiac control complexity through the analysis of the HP series and vascular control complexity through the analysis of the SAP series. The following complexity indices were computed: Shannon’s entropy (SE) [25], the complexity index (CI) [26,27], the normalized complexity index (NCI) [26,27], and sample entropy (SampEn) [28].

SE was conducted in accordance with previous studies by our group [25,27]. Detailed descriptions of the methodology can be found in Porta et al. (2001) [25]. Briefly, stable sequences with HP and SAP values were uniformly quantized over six levels. The original values were replaced by symbols encoding quantization levels from 0 to 5. Then, patterns composed of three consecutive symbols were created. The shape of the distribution of the patterns generated was described by SE [25]. When the distribution is flat, SE is large as all the patterns are equally present and the complexity of the series is high. Meanwhile, when there is a subset of patterns that are most likely associated with the absence or uncommon occurrence of other patterns, SE is small as well as the complexity [25].

The CI and NCI indices are associated with the corrected conditional entropy (CCE) introduced by Porta et al. (1998) [26], grounded on an estimate of conditional entropy (CE). Briefly, the CE measures the amount of information in a new sample that cannot be determined from a sequence of L previous values. The CE computation is biased when computed over a short segment of data, and its calculation was made more robust via the CCE [26]. It has been shown that, as a function of L, the CCE remains constant for white noise, decreases to zero for totally predictable signals, and shows a minimum when repetitive patterns are immersed in noise [27]. The approximation of probabilities through sampling frequencies was performed using the same uniform quantization utilized in the SE analysis. The pattern length L was not a priori fixed, but it was optimized by selecting the value of L minimizing CCE. The CCE minimum was defined as the CI. The NCI was computed by dividing the CI by SE, and, therefore, expresses complexity in terms of dimensionless units [26,27]. Both indices increase with complexity and decrease with regularity.

Finally, the SampEn index was introduced by Richman and Moorman in 2000 [28]. It was calculated using m = 2 and *r* = 0.2·SD. This index monitors how close together a set of patterns is for a few observations [28]. Reduced SampEn values are indicative of greater regularity and predictability.

### 2.6. Statistical Analysis

Initially, given the presence of some individuals with cardiovascular autonomic neuropathy (CAN) in the T2DMG and recognizing the effect of CAN on cardiovascular autonomic modulation, a principal component analysis (PCA) with a Permutational Analysis of Multivariate Variance (PERMANOVA) was performed for T2DMG, including all used variables/indices from the CPET and CRC, to assess the effect of CAN on the results. The presence of CAN was determined through the application of cardiovascular autonomic tests, which were described in detail in previous studies by our group [29]. For the same reason as CAN, the effects of antihypertensive medications were also evaluated using PCA for T2DMG.

Normality was assessed using the Shapiro–Wilk test, and the homogeneity of the data was assessed using the Levene test. Data that did not meet the assumptions were transformed using Box–Cox transformations. Then, a one-way ANOVA was performed to compare the groups (T2DMG and CG), controlling for “age”, “physical activity level” (taking into account the criterion of 150 min of physical activity per week) [30], “metabolic cart”, and “body mass index (BMI)”. This analysis considered all indices and variables from the CPET and CRC. Categorical data were compared using the chi-square test, and the data on the subjects’ characteristics (e.g., blood tests) were evaluated using a *t*-test.

A partial correlation was performed among the VO_2PEAK_ and W/kg_PEAK_ variables and the indices obtained in the CRC analysis for each group (T2DM and CG). This correlation used the “age”, “physical activity level”, and the “metabolic cart” as control variables.

All statistical tests applied a significant threshold of *p* < 0.05. Except for PCA, analyses were performed using the SPSS software, version 25.0. The PCA was performed using the MetaboAnalyst 6.0 software (https://www.metaboanalyst.ca/).

## 3. Results

### 3.1. Participant Characteristics

Initially, a total of 146 individuals were invited to participate in the initial evaluation (anamnesis). However, 33 individuals did not meet the inclusion criteria, and 45 individuals were excluded from this study during the experimental protocol due to significant changes in blood test results (n = 26), dropping out of this study (n = 8), significant changes in blood pressure during data collection (n = 6), and excessive arrhythmias (n = 5). Thus, the data of 68 individuals (34 with T2DM) were analyzed.

Table 1 shows the characteristics of the groups. The T2DMG group was older and had higher values for weight, BMI, leukocytes, glycated hemoglobin (HbA1c), basal insulin, fasting glucose, Homeostasis Model Assessment of Insulin Resistance (HOMA-IR), high-sensitivity C-reactive protein (hs-CRP), very-low-density lipoprotein (VLDL)-cholesterol, and triglycerides. The CG group had higher values for erythrocytes, high-density lipoprotein (HDL)-cholesterol, and low-density lipoprotein (LDL)-cholesterol, although all values were within the normal range or borderline [31]. It is also noteworthy that the T2DMG had more inactive individuals than the CG, and nearly half of its participants had CAN and used antihypertensive medication.

### 3.2. Influence of CAN and Antihypertensive Agents

The PCA showed no influence of CAN on the analyzed data (Figures S1 and S3). This indicated that CAN did not significantly affect the CRC indices and CPET variables’ profiles. Regarding antihypertensive medications, they proved to be slightly more relevant in the PCA analysis (Figures S2 and S3) since antihypertensive agents, unlike CAN, were significant (through the results of PERMANOVA) in comparisons that included principal component 2 (which represents only 23.7% of the total variability of the data). Thus, their effects were previously verified in the partial correlations. However, antihypertensives were particularly related to BMI and, consequently, to W/kg_PEAK_ and VO_2PEAK_ (in mL/kg/min) (Table S1). This information suggests that the association of this type of medication with the data was mainly due to their relationship with BMI. Therefore, adjusting the correlation for antihypertensives could generate artificial data that might not be associated with physiological events. Therefore, antihypertensives were not included as a control variable in the partial correlation analysis, even though they might have a small influence on the results in the SAP series.

### 3.3. Comparison Between Groups

There were no significant differences between the CG and T2DMG in terms of complexity indices (Table 2) after adjusting for BMI, age, metabolic cart, and physical activity level. However, significant differences were observed between the groups for CRF (VO_2PEAK_) and physical fitness (W_PEAK_ and W/kg_PEAK_) variables (Table 2), with higher values in the CG.

### 3.4. Relationship Between CRC and Physical Fitness/CRF

**Figure 1:**
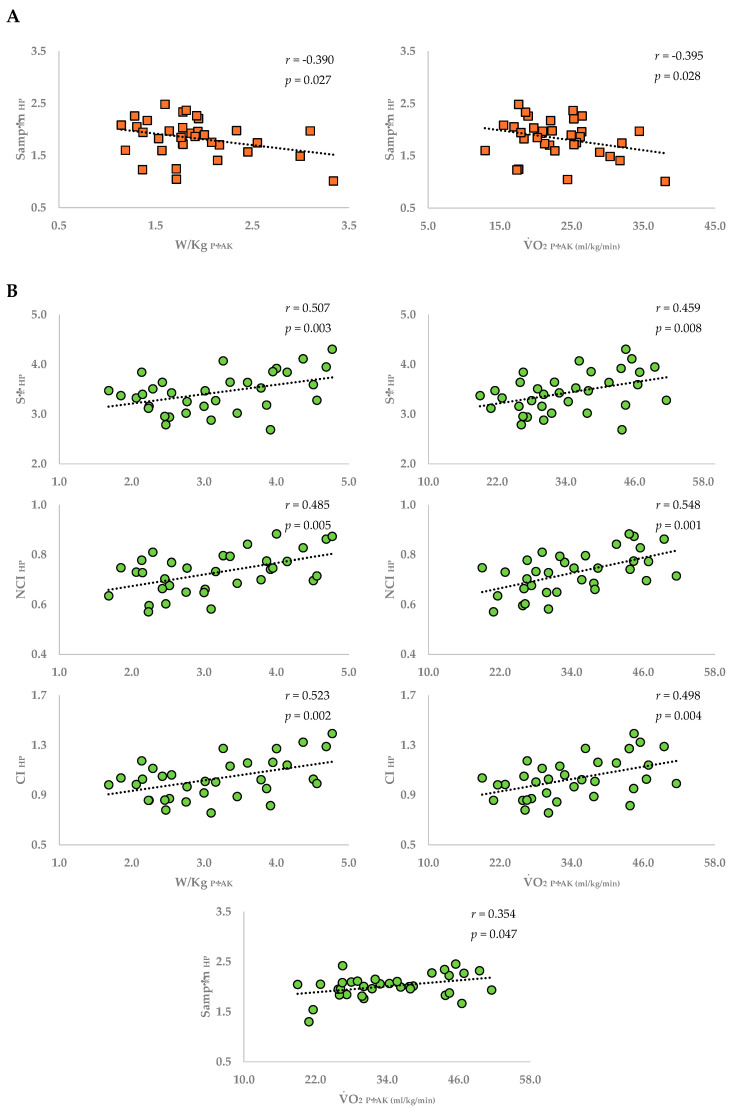
Significant partial correlations. (**A**) Correlations considering T2DMG; (**B**) correlations considering CG. CI_HP_: complexity index for HP series; HP: heart period; NCI_HP_: normalized complexity index for HP series; SampEn_HP_: sample entropy for HP series; SE_HP_: Shannon’s entropy for HP series; T2DMG: type 2 diabetes mellitus group; VO_2PEAK_: peak oxygen consumption; W/kg_PEAK_: relative Watts at peak of exercise. Partial correlations with controls for age, physical activity level, and metabolic cart (the latter only for VO_2PEAK_ in T2DMG, as only in this group were two metabolic carts used), with *p* < 0.05.

**Figure 2:**
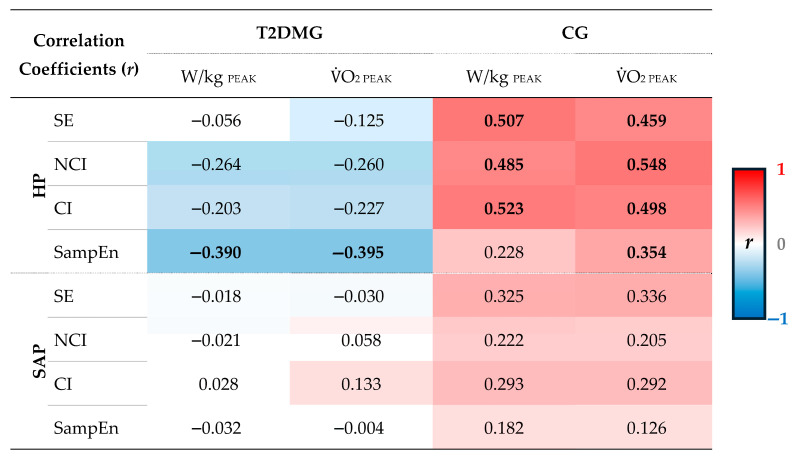
Correlations between CRC indices and physical and cardiorespiratory fitness variables for the CG and T2DMG groups. Bold coefficient (***r***) values are significant (*p* < 0.05). The color intensity is related to the value of the correlation coefficient (**red**: ***r*** = 1; **white**: ***r*** = 0; **blue**: ***r*** = −1). CRC: complexity of cardiovascular regulation; CG: control group; CI: complexity index; HP: heart period; NCI: normalized complexity index; SampEn: sample entropy; SAP: systolic arterial pressure; SE: Shannon’s entropy; T2DMG: type 2 diabetes mellitus group; VO_2PEAK_: peak oxygen consumption (in mL/kg/min); W/kg_PEAK_: relative Watts at peak of exercise. Partial correlations with controls for age, physical activity level, and metabolic cart (the latter only for VO_2PEAK_ in T2DMG, as only in this group were two metabolic carts used), with *p* < 0.05.

After controlling age, physical activity level, and metabolic cart, a significant inverse relationship was only found between SampEn_HP_ and the W/kg_PEAK_ (*r* = −0.390; *p* = 0.027) and VO_2PEAK_ (*r* = −0.395; *p* = 0.028) indices in the T2DMG (Figure 1A). In the CG, only direct proportional relationships were observed between fitness variables and CRC indices: SE_HP_ (W/kg_PEAK_: *r* = 0.507, *p* = 0.003; VO_2PEAK_: *r* = 0.459, *p* = 0.008), NCI_HP_ (W/kg_PEAK_: *r* = 0.485, *p* = 0.005; VO_2PEAK_: *r* = 0.548, *p* = 0.001), CI_HP_ (W/kg_PEAK_: *r* = 0.523, *p* = 0.002; VO_2PEAK_: *r* = 0.498, *p* = 0.004), and SampEn_HP_ (VO_2PEAK_: *r* = 0.354, *p* = 0.047) (Figure 1B). However, no significant SAP series complexity index was observed in both groups. Figure 2 presents all correlation coefficients.

## 4. Discussion

This study investigated the relationship between physical and cardiorespiratory fitness and CRC, considering individuals with T2DM and apparently healthy individuals. As previously observed by our group [13], there were no significant differences in CRC indices values between the evaluated groups, although CRF and physical fitness data were different as expected [32]. The most interesting result, however, was the positive relationship between the complexity indices of the HP variability series and W/kg_PEAK_ and VO_2PEAK_ observed only in apparently healthy individuals.

Despite similar CRC values between individuals with and without T2DM, the relationship between CRC and physical fitness and CRF differed significantly between the groups. This result is counterintuitive because individuals with T2DM should theoretically have a more compromised CRC than healthy individuals, and high physical and cardiorespiratory fitness should independently and positively influence CRC since they are related to health [33,34]. In this context, two plausible explanations may be considered. First, individuals included in T2DM did not present other major diagnosed health complications, such as cardiomyopathy, coronary artery disease, respiratory diseases, or advanced stages of CAN. This could partially explain the similarity between the groups in the present study when CRC indices were compared. Secondly, it has been observed that T2DM may be associated with changes in cardiovascular autonomic modulation without a clear alteration in CRC indices values.

The existing literature provides a small body of research specifically evaluating the association between CRC and diabetes mellitus. Specifically, regarding HP series analysis, the results remain inconclusive and do not provide definitive evidence concerning whether, or how, diabetes mellitus may impact CRC indices values. Some studies indicate a decrease, while others report no significant change [13,35,36,37]. However, it is well-established that T2DM affects cardiac autonomic modulation, as well as the activity and function of the sinoatrial node (SAN) [38,39]. Although a reduction in HP variability due to an impairment in cardiac autonomic modulation is observed in T2DM [29,39], which could result in lower CRC indices values, non-autonomic factors affecting cardiac rhythm must also be considered when analyzing CRC [10,39]. T2DM is linked with a pro-arrhythmogenic metabolic environment [40,41], which, in turn, also leads to alterations in the SAN that compromise intercellular electrical conduction and impact SAN automaticity [38,39]. This overall context implies instabilities within the parasympathetic–SAN–atrial network [38,39,42]. Based on this information, we speculate that the combination of these factors could influence cardiac regulation complexity in individuals with T2DM by increasing the unpredictability of the HP signal unrelated to cardiovascular integrity or parasympathetic autonomic modulation. This could lead to the false impression that CRC is preserved. Accordingly, recent observations indicate that individuals with T2DM exhibit greater heart rate fragmentation (HRF) compared with healthy controls [29]. An elevation in HRF may result in greater HP variability, which could potentially mask the true status related to cardiac autonomic modulation and CRC [42,43]. Considering all these factors, the associations between CRC indices and measures of physical and cardiorespiratory fitness may be changed or abolished. Therefore, CRC does not seem to be positively related to physical and cardiorespiratory fitness in individuals with T2DM who are free of other relevant health complications.

The complexity of the SAP series did not show any significant relationship for either the T2DMG or CG groups. Porta et al. (2012) [24] highlighted that the complexity of the SAP series is lower when compared with the complexity of the HP series. This is attributed to the fact that the HP series is under the influence of autonomic modulation by both branches (sympathetic and parasympathetic), while the SAP series is under the influence of only sympathetic autonomic modulation [24]. Furthermore, a higher sympathetic modulation of the vessels tends to reduce the complexity of the SAP series [24]. In this sense, the absence of significant relationships with physical and cardiorespiratory fitness variables may be attributed, at least in part, to these factors. Therefore, it is not surprising to find out that markers more related to vagal control, such as physical and cardiorespiratory fitness, are more linked to an index such as the CRC of the HP series, more sensitive to vagal withdrawal [24]. However, despite the lack of significance, Figure 2 shows that the CG had solely positive coefficients, whereas the T2DMG had positive and negative coefficients close to zero. This may indicate a trend of a positive association between the complexity of the SAP series and physical and cardiorespiratory fitness in CG, which was not observed in T2DMG. However, it is important to note that some participants in T2DMG were taking antihypertensive medications, which could have slightly influenced these results.

This study provides important information that should be considered when assessing and treating patients with T2DM. Several complexity indices were employed, each derived from distinct calculation methods, thereby providing complementary insights. The SE specifically assesses the distribution of patterns and provides information on the presence or absence of frequent patterns [25]; SampEn assesses the temporal regularity of patterns [28]; and the CI and NCI evaluate the amount of information carried by future values of HP, or SAP, when past data are known [26,27]. Thus, this study conducted a robust analysis of the complexity of HP and SAP series among apparently healthy individuals and those with T2DM. In summary, CRC is not positively related to physical and cardiorespiratory fitness in individuals with T2DM without major health complications, unlike healthy individuals. Although negative correlations were observed between fitness variables and SampEn in the T2DMG, these findings were isolated and among the least significant, which limits reliable inferences. However, the potentially significant influence of non-autonomic factors on CRC in individuals with T2DM should be considered. Finally, the complexity of the SAP series appears to be of little relevance to the association with markers of physical and cardiorespiratory fitness.

This study had some limitations. First, the groups were not similar in terms of age, BMI, or physical activity level. Although these factors were all considered in the statistical analysis, we cannot exclude the possibility that these group differences had a residual influence on the results. Second, two metabolic carts were used. This fact, although controlled by statistical analysis, may have minimally influenced the results. Third, the evaluation was performed on only one sex. Women were not included in this study due to the impact of the menstrual cycle on CRC analyses. It would have been necessary to adjust the evaluations on specific days, and considering our limited resources, collecting data from both sexes was not feasible. In this context, it is important to note that the results of this study are limited to men. Nevertheless, this study employed rigorous criteria for cardiovascular data collection and data analysis, providing valuable new insights into the relationship between CRC and physical and cardiorespiratory fitness variables in the context of T2DM.

## 5. Conclusions

The CRC of the HP series was positively associated with markers of physical and cardiorespiratory fitness in apparently healthy men. However, this relationship was not observed in individuals with T2DM without other serious health complications. In addition, the relationships from the SAP series were insignificant for both CG and T2DMG. These results indicate that further large sample studies are needed to elucidate the factors involved in T2DM that impact CRC. Finally, the relationship evaluated in this study also needs to be elucidated for women.

## Figures and Tables

**Table 1:** Anthropometric and clinical characteristics of the subjects studied.

Characteristics	T2DMG (n = 34)	CG (n = 34)	*p*
Age (years) *	58 (53–63)	51 (48–56)	**<0.001**
Anthropometric data			
Height (m)	1.73 ± 0.06	1.73 ± 0.07	0.794
Body mass (kg) *	86.80 (79.65–92.15)	73.20 (65.50–83.93)	**<0.001**
BMI (kg/m^2^)	28.37 ± 3.05	25.14 ± 2.82	**<0.001**
Physically active (%) **^#^**	32.4	61.8	**0.015**
CAN (n, %)			
Early	35.3	-	-
Definitive	8.8	-	-
Laboratory exams			
Erythrocytes (million/mm^3^) *	4.84 (4.62–5.23)	5.13 (4.95–5.42)	**0.022**
Hemoglobin (g/dL)	14.73 ± 1.43	14.81 ± 0.91	0.770
Hematocrit (%)	42.19 ± 3.73	43.56 ± 2.54	0.391
Leukocytes (mm^3^) *	6700 (6000–7180)	5685 (5103–6865)	**0.007**
HbA1c (%) (mmol/mol) *	7.85 (6.73–9.50)	5.20 (5.00–5.50)	**<0.001**
Basal insulin (mU/mL)	12.59 ± 9.20	6.29 ± 4.30	**<0.001**
Fasting glucose (mg/dL)	173.89 ± 55.72	93.68 ± 6.75	**<0.001**
HOMA-IR	5.23 ± 4.05	1.45 ± 0.97	**<0.001**
hs-CRP (mg/L)	2.27 ± 1.71	1.23 ± 1.07	**0.002**
Total cholesterol (mg/dL)	181.50 ± 43.05	196.79 ± 33.98	0.109
HDL-cholesterol (mg/dL)	43.32 ± 8.98	49.59 ± 11.25	**0.014**
LDL-cholesterol (mg/dL)	105.18 ± 31.03	124.03 ± 30.31	**0.014**
VLDL-cholesterol (mg/dL)	28.85 ± 9.46	23.18 ± 14.92	**0.001**
Triglycerides (mg/dL)	176.18 ± 91.43	117.24 ± 74.60	**<0.001**
Medications			
Hypoglycemic agents (n, %)	88.2	-	-
Antihypertensive agents (n, %)	44.1	-	-
Hypolipidemic agents (n, %)	29.4	-	-
Duration of diabetes (years)	13 ± 8	-	-

* Data presented as medians and interquartile ranges (Mann–Whitney test). ^#^ Physically active: ≥150 min of physical activity per week (comparison evaluated by the chi-square test). Bold values are significant. The BMI, basal insulin, Homa-IR, hs-CRP, VLDL, and triglycerides were analyzed using the transformed data (original data are presented in the table for clarity and interpretation). BMI: body mass index; CAN: cardiovascular autonomic neuropathy; HbA1c: glycated hemoglobin; HDL: high-density lipoprotein; HOMA-IR: Homeostasis Model Assessment of Insulin Resistance; hs-CRP: high-sensitivity C-reactive protein; LDL: low-density lipoprotein; VLDL: very-low-density lipoprotein. *T*-test with *p* < 0.05.

**Table 2:** Differences in CRF and physical fitness between the CG and T2DMG.

Variable	T2DMG (n = 34)	CG (n = 34)	*p*	*d* (95% CI) *
CPET				
W_PEAK_	158.56 ± 38.94	233.00 ± 60.46	**0.008**	0.67 (0.18; 1.16)
W/kg_PEAK_	1.89 ± 0.52	3.15 ± 0.90	**<0.001**	1.16 (0.64; 1.68)
VO_2PEAK_ (mL/min)	1961.60 ± 481.88	2543.01 ± 647.87	**<0.001**	1.10 (0.58; 1.62)
VO_2PEAK_ (mL/kg/min)	23.19 ± 5.68	34.24 ± 8.98	**<0.001**	1.35 (0.82; 1.88)
HR_PEAK_ (bpm)	147.56 ± 17.38	160.88 ± 15.17	0.243	0.14 (−0.34; 0.62)
VO_2_/HR (mL/bpm)	13.30 ± 2.92	15.82 ± 3.78	**0.001**	0.81 (0.30; 1.32)
CRC				
CI_HP_	1.00 ± 0.18	1.03 ± 0.16	0.611	0.19 (−0.27; 0.67)
CI_SAP_	0.90 ± 0.12	0.91 ± 0.15	0.699	0.16 (−0.32; 0.64)
NCI_HP_	0.67 ± 0.12	0.73 ± 0.08	0.484	0.22 (−0.26; 0.70)
NCI_SAP_	0.58 ± 0.08	0.60 ± 0.08	0.612	0.26 (−0.22; 0.74)
SampEn_HP_	1.83 ± 0.36	2.01 ± 0.24	0.078	0.44 (−0.04; 0.92)
SampEn_SAP_	1.65 ± 0.30	1.64 ± 0.29	0.906	0.03 (−0.45; 0.51)
SE_HP_	3.45 ± 0.38	3.43 ± 0.40	0.169	0.35 (−0.13; 0.83)
SE_SAP_	3.37 ± 0.27	3.29 ± 0.35	0.227	0.30 (−0.18; 0.78)

* Calculated based on adjusted mean differences. Bold values are significant. Only the HR_PEAK_ was analyzed using the transformed data (original data are presented in the table for clarity and interpretation). *d*: Cohen’s *d*; 95% CI: 95% confidence interval; CG: control group; CI_HP_: complexity index for HP series; CI_SAP_: complexity index for SAP series; CPET: cardiopulmonary exercise test; CRC: cardiovascular regulation complexity; CRF: cardiorespiratory fitness; HP: heart period; HR_PEAK_: peak heart rate; NCI_HP_: normalized complexity index for HP series; NCI_SAP_: normalized complexity index for SAP series; SampEn_HP_: sample entropy for HP series; SampEn_SAP_: sample entropy for SAP series; SAP: systolic arterial pressure; SE_HP_: Shannon’s entropy for HP series; SE_SAP_: Shannon’s entropy for SAP series; T2DMG: type 2 diabetes mellitus group; VO_2_/HR: oxygen pulse; VO_2PEAK_: peak oxygen consumption; W_PEAK_: Watts at peak of exercise; W/kg_PEAK_: relative Watts at peak of exercise. One-way ANOVA with controls for age, BMI, physical activity level, and metabolic cart (the latter only for VO_2_ variables), with *p* < 0.05.

## Data Availability

The original contributions presented in this study are included in this article/Supplementary Material. Further inquiries can be directed to the corresponding authors.

## Supplementary Materials

The following supporting information can be downloaded at https://www.mdpi.com/article/10.3390/healthcare14070940/s1, Table S1: Partial correlations considering individuals with T2DM. Figure S1. Principal components analysis for CAN. Figure S2. Principal components analysis for antihypertensive agents. Figure S3. Scree plot.

## Abbreviations

The following abbreviations are used in this manuscript:

BMI	Body mass index
CAN	Cardiovascular autonomic neuropathy
CE	Conditional entropy
CCE	Corrected CE
CG	Control group
CI	Complexity index
CPET	Cardiopulmonary exercise test
CRC	Cardiovascular regulation complexity
CRF	Cardiorespiratory fitness
ECG	Electrocardiogram
HbA1c	Glycated hemoglobin
HDL	High-density lipoprotein
HOMA-IR	Homeostasis Model Assessment of Insulin Resistance
HP	Heart period
HRF	Heart rate fragmentation
hs-CRP	High-sensitivity C-reactive protein
LDL	Low-density lipoprotein
NCI	Normalized complexity index
PCA	Principal component analysis
SampEn	Sample entropy
SAP	Systolic arterial pressure
SE	Shannon’s entropy
T2DM	Type 2 diabetes mellitus
T2DMG	Type 2 diabetes mellitus group
VLDL	Very-low-density lipoprotein
VO_2PEAK_	Peak oxygen consumption
VO_2_/HR	Oxygen pulse
W_PEAK_	Peak power
W/kg_PEAK_	Relative Watts at peak of exercise
