# Supplementary material for: Complexity of Cardiovascular Regulation and Its Association with Physical and Cardiorespiratory Fitness in Men with Type 2 Diabetes Mellitus
Source: Healthcare (Basel). 2026 Apr 3;14(7):940. doi: 10.3390/healthcare14070940 (PMC13073779; doi:10.3390/healthcare14070940)
Supplement: Supplementary file 1 [file healthcare-14-00940-s001.zip › healthcare-4202718-supplementary.pdf]

Supplementary material

Complexity of Cardiovascular Regulation and Its Association with Physical and Cardiorespiratory Fitness in Men with Type 2 Diabetes Mellitus

Étore De F. Signini[1]*, Raphael M. de Abreu[2,3], Alex Castro[4], Andréia M. Santos[1], Gabriela A. M. Galdino[1], Silvia C. G. Moura[1], Stephanie N. Linares[1], Juliana C. Milan-Mattos[1], Rafaella M. Zambetta[1], Alberto Porta[5,6] and Aparecida M. Catai[1]

1. Department of Physiotherapy, Universidade Federal de São Carlos, São Carlos 13565-905, Brazil; andreia.machado@estudante.ufscar.br (A.M.S.); gabimgaldino@hotmail.com (G.A.M.G.); silvinhacgmoura@gmail.com (S.C.G.M.); stenolinares@gmail.com (S.N.L.); julianacristinamilan@gmail.com (J.C.M.-M.); rafa.m.zambetta@gmail.com (R.M.Z.)
2 Department of Health, LUNEX University of Applied Sciences, 4671 Differdange, Luxembourg; rmartinsdeabreu@lunex.lu
3 LUNEX ASBL Luxembourg Health & Sport Sciences Research Institute, 4671 Differdange, Luxembourg
4 Brazilian Biosciences National Laboratory, Brazilian Center for Research in Energy and Materials, Campinas 13083-100, Brazil; ax.castro@yahoo.com.br
5 Department of Biomedical Sciences for Health, University of Milan, 20133 Milan, Italy; alberto.porta@unimi.it
6 Department of Cardiothoracic, Vascular Anesthesia and Intensive Care, IRCCS Policlinico San Donato, 20097 San Donato Milanese, Italy
* Correspondence: signinietof@gmail.com (É.D.F.S.); mcatai@ufscar.br (A.M.C.);
Tel.: +55-16-33518705 (É.D.F.S.); Fax: +55-16-33612081 (É.D.F.S.)

Table S1. Partial correlations considering individuals with T2DM.

Figure S1. Principal components analysis for CAN.

Figure S2. Principal components analysis for antihypertensive agents.

Figure S3. Scree plot.

Table S1. Partial correlations considering individuals with T2DM.

**Indices/Variables**		BMI (kg/m2)	W	W/kg	$\dot{V}O_2$ (ml/kg/min)	$\dot{V}O_2$ (ml/kg/min)	SE HP	SE SAP	NCI HP	CI HP	NCI SAP	CI SAP	SampEn HP	SampEn SAP
BMI (kg/m2)	***r***	1.000	-0.242	**-0.580**	-0.314	0.063	-0.150	0.198	-0.029	-0.088	0.246	0.217	0.203	0.257
	p		0.169	**<0.001**	0.071	0.725	0.397	0.261	0.872	0.620	0.162	0.218	0.249	0.143
W	***r***	-0.242	1.000	**0.894**	**0.883**	**0.862**	-0.181	-0.056	-0.203	-0.280	0.075	0.060	-0.267	0.086
	p	0.169		**<0.001**	**<0.001**	**<0.001**	0.305	0.753	0.250	0.109	0.674	0.735	0.127	0.629
W/kg	***r***	**-0.580**	**0.894**	1.000	**0.881**	**0.655**	-0.071	-0.185	-0.217	-0.198	-0.084	-0.083	-0.314	-0.080
	p	**<0.001**	**<0.001**		**<0.001**	**<0.001**	0.688	0.296	0.217	0.262	0.637	0.639	0.070	0.652
$\dot{V}O_2$ (ml/kg/min)	***r***	-0.314	**0.883**	**0.881**	1.000	**0.892**	-0.217	0.023	-0.248	-0.311	0.056	0.117	-0.298	0.062
	p	0.071	**<0.001**	**<0.001**		**<0.001**	0.218	0.897	0.157	0.074	0.754	0.510	0.087	0.729
$\dot{V}O_2$ (ml/min)	***r***	0.063	**0.862**	**0.655**	**0.892**	1.000	-0.314	0.165	-0.216	**-0.368**	0.205	0.260	-0.223	0.217
	p	0.725	**<0.001**	**<0.001**	**<0.001**		0.071	0.351	0.220	**0.032**	0.244	0.138	0.205	0.217
SE HP	***r***	-0.150	-0.181	-0.071	-0.217	-0.314	1.000	-0.110	**0.554**	**0.902**	-0.125	-0.154	**0.524**	-0.018
	p	0.397	0.305	0.688	0.218	0.071		0.536	**0.001**	**<0.001**	0.481	0.383	**0.001**	0.921
SE SAP	***r***	0.198	-0.056	-0.185	0.023	0.165	-0.110	1.000	0.096	-0.044	**0.696**	**0.852**	0.133	**0.668**
	p	0.261	0.753	0.296	0.897	0.351	0.536		0.589	0.807	**<0.001**	**<0.001**	0.452	**<0.001**
NCI HP	***r***	-0.029	-0.203	-0.217	-0.248	-0.216	**0.554**	0.096	1.000	**0.823**	0.062	0.008	**0.811**	0.151
	p	0.872	0.250	0.217	0.157	0.220	**0.001**	0.589		**<0.001**	0.726	0.964	**<0.001**	0.395
CI HP	***r***	-0.088	-0.280	-0.198	-0.311	**-0.368**	**0.902**	-0.044	**0.823**	1.000	-0.088	-0.117	**0.694**	-0.006
	p	0.620	0.109	0.262	0.074	**0.032**	**<0.001**	0.807	**<0.001**		0.621	0.509	**<0.001**	0.972
NCI SAP	***r***	0.246	0.075	-0.084	0.056	0.205	-0.125	**0.696**	0.062	-0.088	1.000	**0.925**	0.108	**0.730**
	p	0.162	0.674	0.637	0.754	0.244	0.481	**<0.001**	0.726	0.621		**<0.001**	0.541	**<0.001**
CI SAP	***r***	0.217	0.060	-0.083	0.117	0.260	-0.154	**0.852**	0.008	-0.117	**0.925**	1.000	0.028	**0.709**
	p	0.218	0.735	0.639	0.510	0.138	0.383	**<0.001**	0.964	0.509	**<0.001**		0.876	**<0.001**
SampEn HP	***r***	0.203	-0.267	-0.314	-0.298	-0.223	**0.524**	0.133	**0.811**	**0.694**	0.108	0.028	1.000	0.229
	p	0.249	0.127	0.070	0.087	0.205	**0.001**	0.452	**<0.001**	**<0.001**	0.541	0.876		0.192
SampEn SAP	***r***	0.257	0.086	-0.080	0.062	0.217	-0.018	**0.668**	0.151	-0.006	**0.730**	**0.709**	0.229	1.000
	p	0.143	0.629	0.652	0.729	0.217	0.921	**<0.001**	0.395	0.972	**<0.001**	**<0.001**	0.192	
Age (Years)	***r***	-0.079	**-0.388**	**-0.341**	-0.201	-0.194	-0.026	0.319	0.239	0.075	0.026	0.121	0.139	0.171
	p	0.656	**0.024**	**0.049**	0.254	0.272	0.884	0.066	0.174	0.674	0.883	0.494	0.433	0.335
Metabolic Cart	***r***	-0.051	0.235	0.170	**0.355**	**0.390**	-0.155	**0.411**	-0.126	-0.201	0.159	0.250	-0.050	0.206
	p	0.773	0.181	0.336	**0.039**	**0.023**	0.380	**0.016**	0.479	0.254	0.369	0.155	0.778	0.242
Physically active	***r***	-0.246	0.282	0.294	0.219	0.180	-0.097	-0.160	0.273	0.025	-0.172	-0.192	0.214	0.034
	p	0.161	0.107	0.092	0.213	0.309	0.584	0.366	0.118	0.889	0.332	0.276	0.223	0.850
Antihypertensive	***r***	**0.440**	-0.336	**-0.446**	**-0.346**	-0.199	0.035	**0.390**	0.331	0.187	0.240	0.307	**0.433**	0.274
	p	**0.009**	0.052	**0.008**	**0.045**	0.259	0.843	**0.023**	0.056	0.291	0.171	0.077	**0.011**	0.117

Bold values are significant. Variables in italics are covariates. The correlations presented are not adjusted for covariates. **When adjusting the values for the use of antihypertensive drugs, no significant association was observed between CRC indices and fitness variables.** CI HP: complexity index for HP series; CI SAP: complexity index for SAP series; CRC: cardiovascular regulation complexity; HP: heart period; NCI HP: normalized complexity index for HP series; NCI SAP: normalized complexity index for SAP series; SampEn HP: sample entropy for HP series; SampEn SAP: sample entropy for SAP series; SAP: systolic arterial pressure; SE HP: Shannon's entropy for HP series; SE SAP: Shannon's entropy for SAP series; $\dot{V}O_2$ PEAK: peak oxygen consumption; W/kg PEAK: relative Watts at peak of exercise. Partial correlations with no adjustments, with $p < 0.05$.

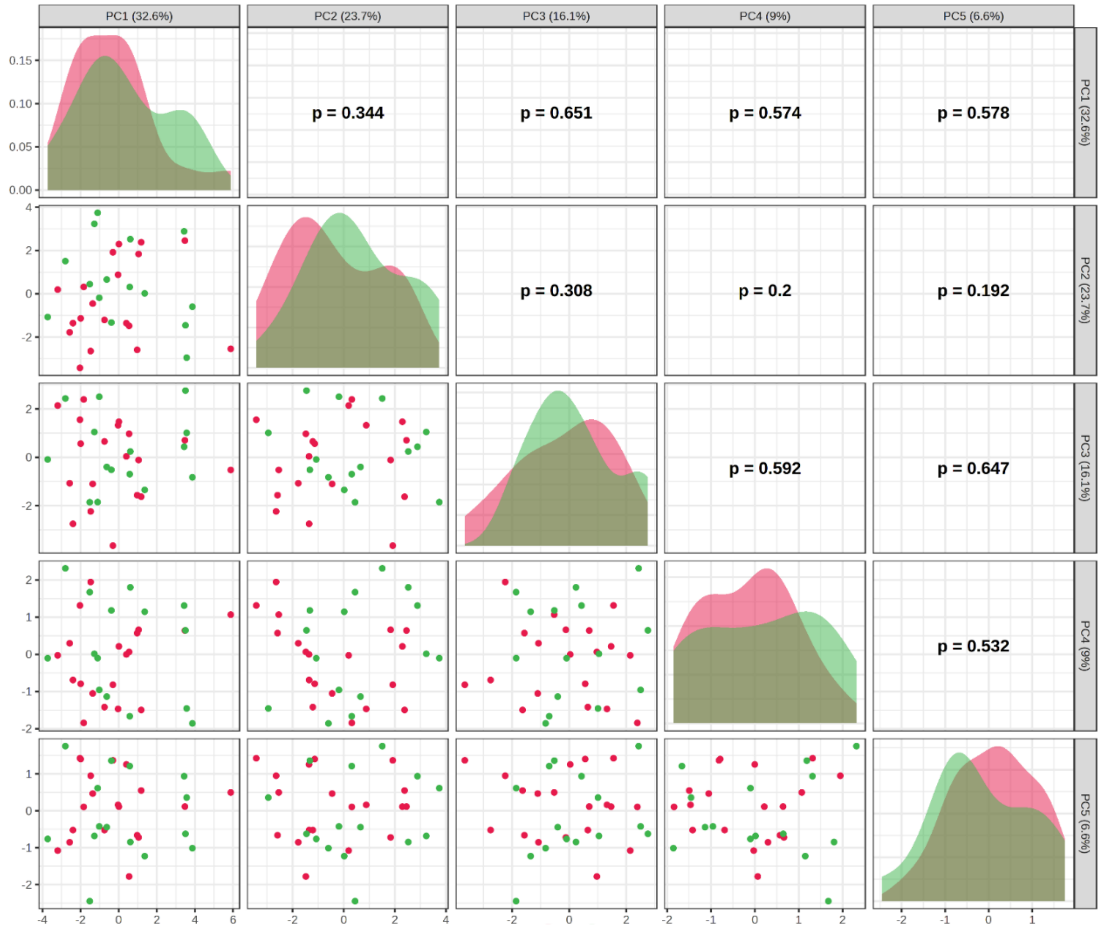

Figure S1. Principal components analysis for CAN. The figure shows the score plot for the first five principal components, which were defined using the criterion of eigenvalues > 1. The analysis included all indices and variables contained in Table 2, as well as age and BMI. **Red dots represent individuals without CAN, and green dots represent individuals with CAN**. The overlap of the groups indicates that there is no difference between them (the *p*-values of each principal component comparison were greater than 0.05 in PERMANOVA) when considering all the included variables (the entire profile). BMI: body mass index; CAN: cardiovascular autonomic neuropathy; PC: principal component. Data processed in *MetaboAnalyst 6.0* software (https://www.metaboanalyst.ca/).

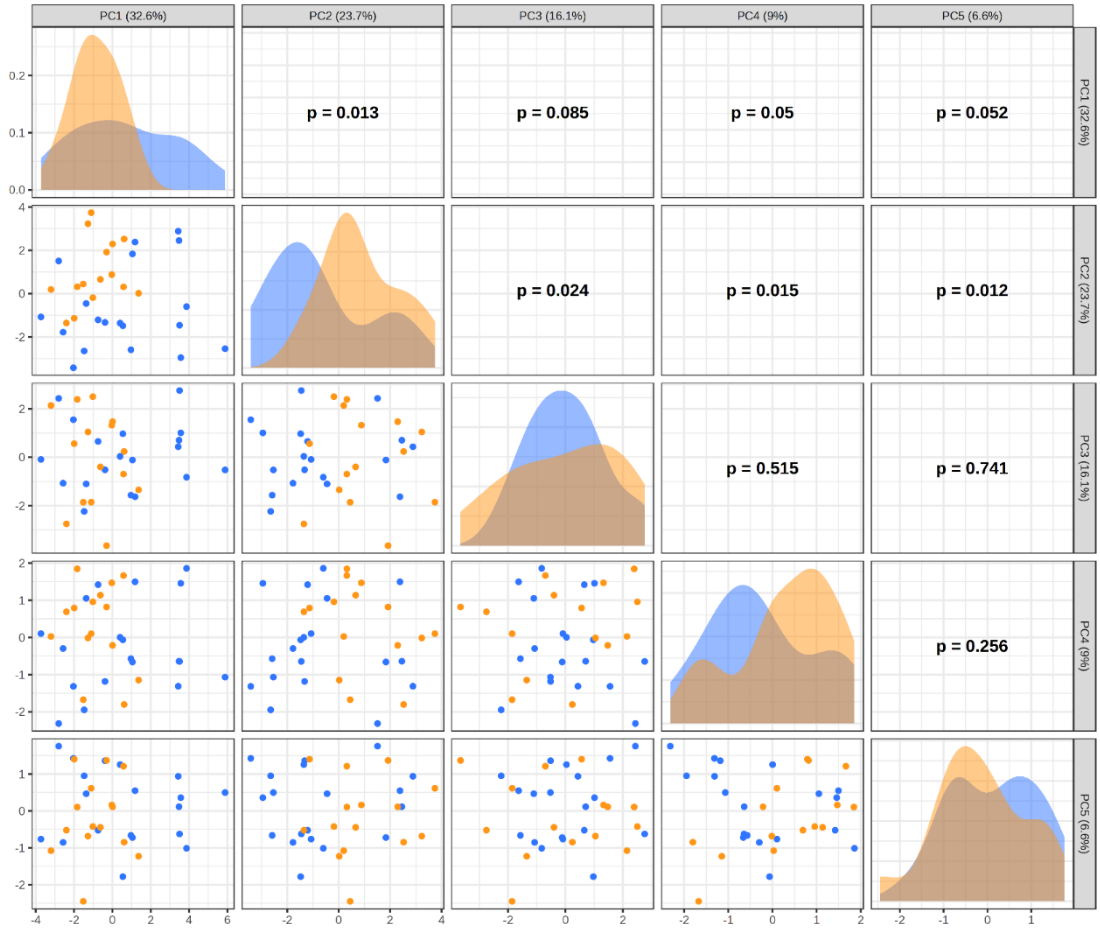

Figure S2. Principal components analysis for antihypertensive agents. The figure shows the score plot for the first five principal components, which were defined using the criterion of eigenvalues > 1. The analysis included all indices and variables contained in Table 2, as well as age and BMI. **Blue dots represent individuals without using antihypertensive agents, and orange dots represent individuals using antihypertensive agents**. There is an overlap between the groups. However, individuals who use antihypertensive medication show less variation among themselves. They also indicate a tendency for separation in PCs 1 and 2, especially. Considering this information and the existence of significant *p*-values among some principal component comparisons ($p < 0.05$ in PERMANOVA), individuals using antihypertensive medication have slightly different profiles than those not using antihypertensive medication, when considering all the included variables (the entire profile). BMI: body mass index; PC: principal component. Data processed in *MetaboAnalyst 6.0* software (https://www.metaboanalyst.ca/).

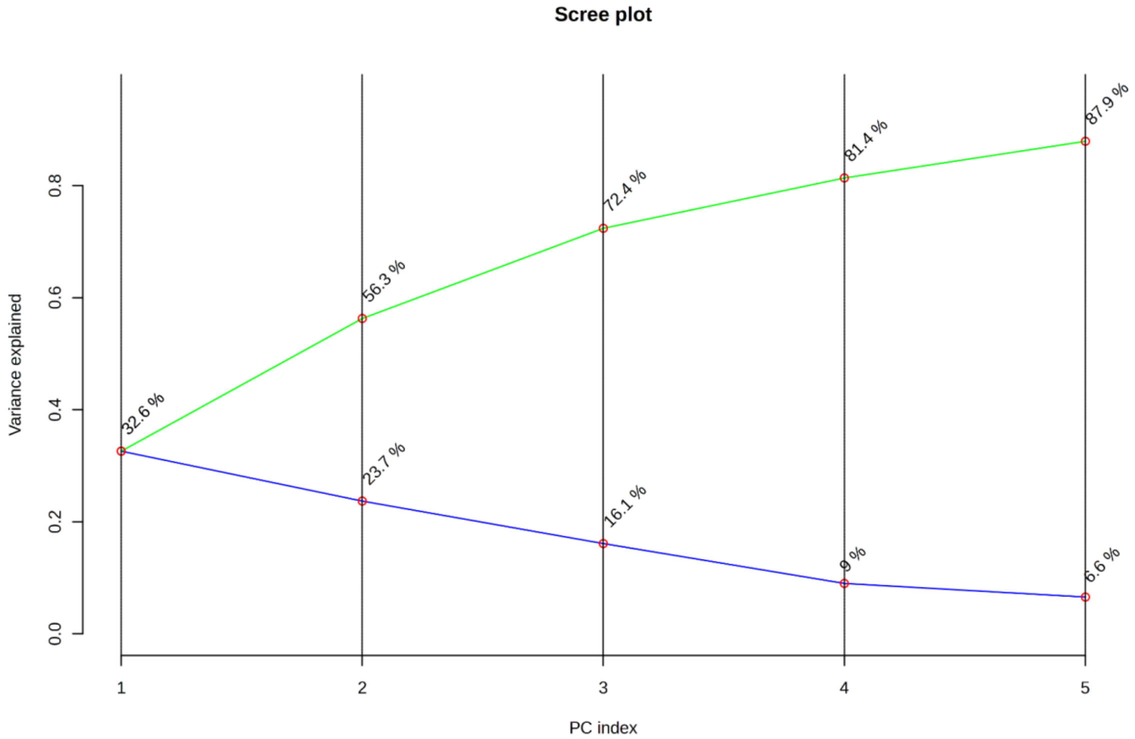

Figure S3. Scree plot. The use of 5 principal components (eigenvalue > 1) explains a total of 87.9% of the total variability of the data. Data processed in *MetaboAnalyst 6.0* software (https://www.metaboanalyst.ca/).
